# Temporal Bone Cholesteatoma: Typical Findings and Evaluation of Diagnostic Utility on High Resolution Computed Tomography

**DOI:** 10.7759/cureus.22730

**Published:** 2022-03-01

**Authors:** Sameeh Uz Zaman, Varsha Rangankar, Krishnarjun Muralinath, Viraj Shah, Gowtham K, Rishikesh Pawar

**Affiliations:** 1 Radiology, Dr. D. Y. Patil Medical College, Hospital & Research Centre, Pune, IND; 2 Otolaryngology - Head and Neck Surgery, Dr. D. Y. Patil Medical College, Hospital & Research Centre, Pune, IND

**Keywords:** chronic suppurative otitis media, external auditory canal cholesteatoma, hrct temporal bone, high resolution computed tomography, cholesteatoma

## Abstract

Background

Pre-operative assessment of middle ear cholesteatoma is a must for assessing the disease's location, extent, and complication, and high-resolution computed tomography (HRCT) is the modality of choice. Therefore, this study aims to assess the common signs of cholesteatoma on HRCT and its diagnostic ability.

Methods

Fifty patients with suspected cholesteatoma were considered for the study, which was carried out on an Ingenuity Core 128 slice CT scanner (Philips, Amsterdam, Netherlands). The bilateral temporal bones of 50 patients were assessed for soft tissue density and associated findings. The number of temporal bones with soft tissue density was then correlated with intra-operative and histopathological examinations (HPE).

Results

Out of 100 temporal bones, 63 were diseased, and 37 were normal temporal bones. Epitympanum/Prussak's space was the most involved site with soft tissue density seen in 60/63 (95.2%) diseased temporal bones, followed by aditus ad antrum and mesotympanum, which was seen in 51/63 (80.9%) diseased temporal bones. The majority of the soft tissue lesions were non-dependent, accounting for 43/63 (68.2%) of the diseased temporal bones. Bony erosions were seen in 54/63 (85.7%) and bony expansion in 35/63 (55.5%) of the diseased temporal bones. HRCT showed a sensitivity of 100%, specificity of 88.1%, a positive predictive value (PPV) of 92.1%, a negative predictive value (NPV) of 100%, and accuracy of 95% for detection of cholesteatoma.

Conclusion

HRCT of the temporal bone precisely demonstrates cholesteatoma's location, extent, and bony changes. Therefore, it has exceptional sensitivity, high specificity, and accuracy in diagnosing cholesteatoma.

## Introduction

Chronic otitis media (COM) is a stage of ear disease in which there is a long-term infection of the middle ear cleft, which includes the Eustachian tube, the middle ear, and the mastoid. In this stage, there is the presence of a ruptured tympanic membrane and drainage [1]. Clinically, there are two main types of chronic suppurative otitis media: one with no cholesteatoma, known as the "safe" type, and one with cholesteatoma, known as the "unsafe" type [2].

Cholesteatoma is, in simple terms, "skin in the wrong place." Cholesteatoma consists of an outer lining composed of stratified squamous epithelium, an inner keratin debris content within the cholesteatoma sac which is in turn secreted by the epithelium, and an external peri-matrix that secretes bone destroying proteolytic enzymes [3].

The clinical diagnosis of cholesteatoma is made by otoscopic examination, and the gold standard is histopathology or during operative exploration. On the other hand, imaging measures for diagnosis, such as high-resolution computed tomography (HRCT) and magnetic resonance imaging (MRI), are utilized. HRCT is the imaging modality of choice for diagnosing cholesteatoma due to its ability to assess the gross extent and erosion of the fine bony architecture of the temporal bone. Hence, HRCT is an indispensable pre-operative necessity [3].

On HRCT, cholesteatoma appears as a soft-tissue "mass-like" density in the middle ear cavity and mastoid antrum with associated signs of mass effect in the form of surrounding smooth bony erosions and expansion cavities [4]. The likelihood of cholesteatoma increases when the soft tissue opacity is non-dependent [5]. The absence of soft tissue density in the middle ear mastoid complex rules out cholesteatoma. However, HRCT cannot characterize the soft tissue density and differentiate cholesteatoma from inflammatory/granulation tissue or scar tissue [6]. The purpose of the present study is to enumerate the typical signs of cholesteatoma on HRCT and evaluate the diagnostic ability of HRCT for temporal bone cholesteatoma.

## Materials and methods

Case selection

The present study was a descriptive observational research between September 2019 and August 2021. It was conducted after permission was granted from the Institutional Ethics Sub-Committee (IESC) at Dr. D.Y. Patil Medical College, Hospital and Research Center, Pune, MH, India, with approval number IESC/PGS/2019/173. Every patient took written informed consent. Fifty patients were referred from the Otorhinolaryngology and Head and Neck Surgery department with suspicion of primary cholesteatoma or recurrent cholesteatoma in post-operative cases based on clinical presentation, otoscopic examination and audiometric tests. Patients of all ages were included in our study. The patients with soft tissue density in the temporal bone suspected to be cholesteatoma on HRCT underwent surgery. Surgery was performed by two otorhinolaryngologists with experience of 15 and 16 years respectively. The final diagnosis was arrived at utilizing surgical and histopathological validation.

Imaging technique

HRCT imaging was obtained on Philips Ingenuity Core (Philips, Amsterdam, Netherlands), a 128 slice CT scanner. The high-resolution CT images were obtained with 20 × 0.625 collimation and 0.8 mm thickness using 320 mAs, and 120kVp with ultrathin image reconstruction using the high-resolution bone algorithm in the axial plane with 0.5 mm section thickness, 0.01 mm increments, and a FOV of 100, with a matrix size of 512 × 512. This isotropic image data was used to obtain coronal and sagittal reformatted images. The image interpretation was made using a 3D workstation.

Imaging evaluation

In our 50-patient sample, the bilateral temporal bones of each patient were assessed. Total 100 high-resolution temporal bone studies of the 50 patients were evaluated in detail in axial and reformatted coronal and sagittal planes. The HRCT findings in both the temporal bones were noted under the headings: aeration status of mastoid, sclerosis of mastoid air cells, location of the soft tissue lesion, the extent of soft tissue lesion, bony expansion (e.g., widened aditus ad antrum, mastoid cavity), bony erosions (e.g., scutum, middle ear walls, mastoid air cells), ossicular chain status (malleus, incus, stapes), tegmen tympani erosion, sinus plate erosion, facial canal dehiscence, and other findings. All the HRCT studies were assessed by a single radiologist with an experience of 16 years with special focus on neuroradiology and head and neck imaging.

Statistical analysis

Sensitivity, specificity, positive predictive value (PPV), negative predictive value (NPV), and accuracy of HRCT in diagnosing cholesteatoma were calculated with a 95% confidence interval by correlating them to the gold standard-HPE/Intraoperative results. The statistical analysis was done using Microsoft Excel 2007 and Statistical Package for Social Sciences (SPSS) version 26 (IBM Corp., Armonk, NY, USA).

## Results

The 50 patients with chronic suppurative otitis media (CSOM) or probable post-operative cholesteatoma recurrence included in this research had an average age of 25±10.8, ranging from eight to 65 years. The majority of patients (20%) were aged between 30 and 40 years. There was an equal number of males and females (25 patients each), with no gender preference. Our research sample's most common clinical symptom was purulent ear discharge, found in 42 cases (84%). Hearing loss (64%) and otalgia were common complaints (46%). Most of the patients (60%) had history of recurrent upper respiratory tract infection. Only one patient had a history of ear trauma.

On HRCT, 35 patients (70%) had soft tissue density in the unilateral temporal bone, and 14 patients (28%) had soft tissue density in bilateral temporal bones.

In our study, the bilateral temporal bones of each patient, i.e., 100 temporal bones in 50 patients, were assessed on HRCT. The temporal bones that showed soft tissue density was labeled as diseased temporal bone on HRCT. Out of 100 temporal bones, 63 were diseased, and 37 were normal temporal bones. Of the 63 diseased temporal bones, 54 were primary diseased, and nine had recurrent disease post-operatively. One clinically symptomatic patient had no soft tissue on HRCT.

Epitympanum/Prussak's space was the most involved site with soft tissue density seen in 60/63 (95.2%) diseased temporal bones, followed by aditus ad antrum and mesotympanum, which was seen in 51/63 (80.9%) diseased temporal bones. Soft tissue density was present in the mastoid antrum and air cells in 46/63 (73%) diseased temporal bones and hypotympanum in 20/63 (31.7%) diseased temporal bones. External auditory canal (EAC), sinus tympani, facial canal recess, and eustachian tube were the least involved in 10/63 (15.8%), 5/63 (7.9%), 4/63 (6.3%), and 1/63 (1.5%) diseased temporal bones, respectively (Table 1).

**Table 1:** Soft tissue density at different locations in diseased temporal bones on HRCT EAC: external auditory canal, HRCT: high-resolution computed tomography

Involvement of individual parts	Number	Percentage
Epitympanum/Prussak space	60	95.2
Mesotympanum	51	80.9
Hypotympanum	20	31.7
Aditus ad Antrum	51	80.9
Mastoid Antrum and air cells	46	73
Sinus Tympani	5	7.9
Facial canal recess	4	6.3
Eustachian Tube	1	1.5
EAC	10	15.8

**Figure 1:**
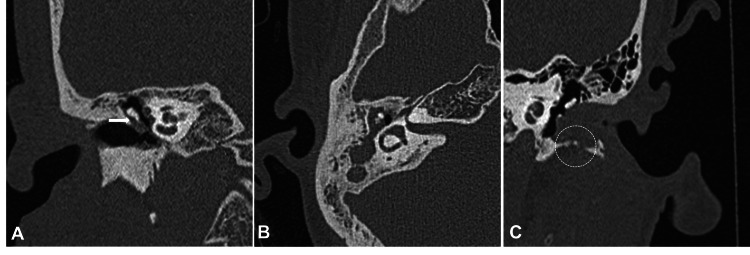
HRCT images demonstrating locations of cholesteatoma. Coronal reformatted HRCT image of the right temporal bone (A) shows non-dependent soft tissue in Prussak’s space (white arrow) proved as cholesteatoma. Axial HRCT image of right temporal bone showing attico-antral cholesteatoma (B). Coronal reformatted HRCT image demonstrating soft tissue density lesion and bony erosions in the left EAC (C), a rare cholesteatoma location. EAC: external auditory canal, HRCT: high-resolution computed tomography

The most common typical site (Table 2) of soft tissue density involvement was attico-antral in 32/63 (50.7%) diseased temporal bones, followed by extensive holotympanic seen in 19/63 (30.1%) and attic in 10/63 (15.8%) diseased temporal bones (Figure 1A, 1B). Conversely, the least common locations were isolated mesotympanum and EAC (Figure 1C), each seen in 1/63 (1.5%) diseased temporal bone. 

**Table 2:** Typical locations of soft tissue density in the diseased temporal bones on HRCT. EAC: external auditory canal, HRCT: high-resolution computed tomography

Involvement	Number	Percentage
Attico-antral	32	50.7
Holotympanic	19	30.1
Attic	10	15.8
Mesotympanum only	1	1.5
EAC	1	1.5

**Figure 2:**
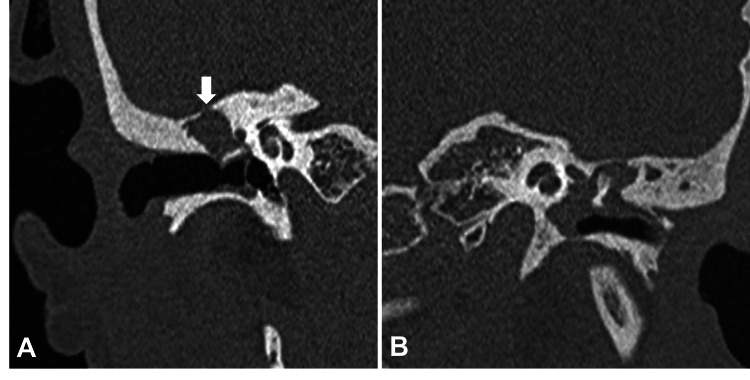
A reformatted coronal CT image of a patient which shows a non-dependent soft tissue density in the right middle ear (A) which is causing erosion of the tegmen tympani (white arrow). A coronal reformatted CT image of a patient which shows a soft tissue density in the left middle ear (B) which is causing erosion and deossification of the head of malleus.

**Figure 3:**
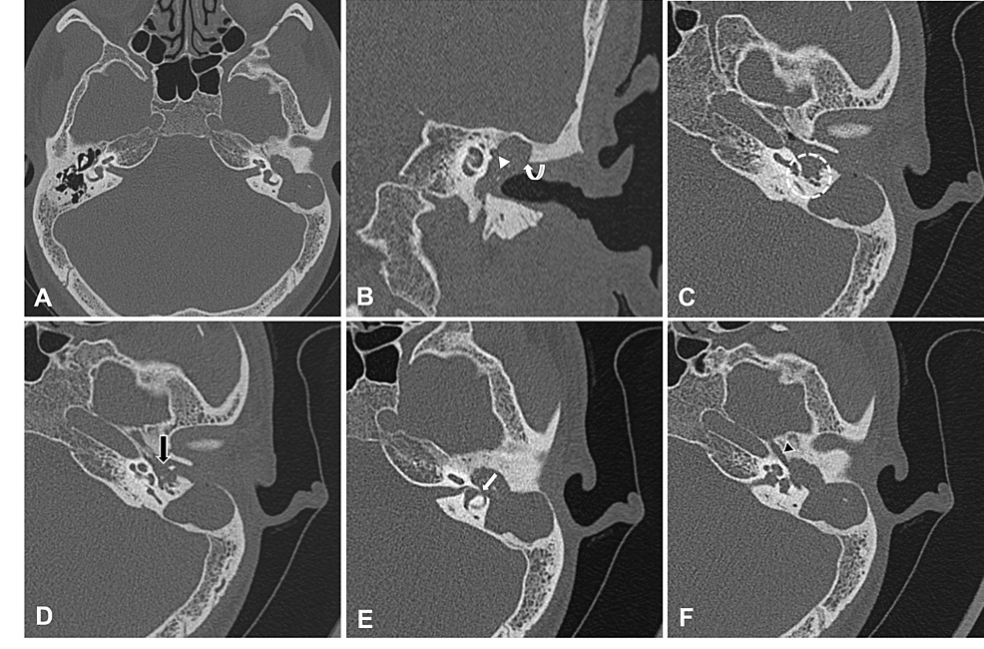
Axial HRCT image (A) showing a large well-defined soft tissue density in the left middle ear extending into the aditus and mastoid antrum, causing bony expansion and destruction of the malleus and incus with loss of ice cream cone appearance. Coronal reformatted CT image in the same patient (B) shows soft tissue density occupying the epitympanum, mesotympanum, and hypotympanum. It is seen causing erosion of the scutum (curved arrow) and inferior wall of the horizontal portion of the facial canal (arrowhead). Axial HRCT image (C) showing soft tissue (encircled) is seen extending into the sinus tympani and facial canal recesses. Axial HRCT image showing the destruction of stapes (D) and mastoid cortex dehiscence with thinning of dural sinus plate (D). Axial HRCT image (E) shows a focal defect in the wall of the lateral semicircular canal (white arrow) suggestive of perilymphatic fistula. Soft tissue extension into the eustachian tube (black arrowhead) was also seen (F). HRCT: high-resolution computed tomography

The most consistent imaging finding of CSOM was the loss of aeration and sclerosis of mastoid air cells and was present in 61/63 (96.8%) diseased temporal bones. The majority of the soft tissue lesions were non-dependent (Figure 2A), accounting for 43/63 (68.2%) of the soft tissue density lesions in the diseased temporal bones. The other common findings suggesting CSOM with cholesteatoma were bony erosions seen in 54/63 (85.7%) diseased temporal bones followed by bony expansion (Figure 3A) in 35/63 (55.5%) of the diseased temporal bones. Lateral semicircular fistula (Figure 3E) and petrous apicitis were present in 3/63 (4.7%) and 1/63 (1.5%) of the diseased temporal bones (Table 3). Incus was the most common structure eroded, seen in 38/63 (63%) of the diseased temporal bones. The other common structures eroded were malleus (Figure 2B) seen in 37/63 (58.7%), scutum (Figure 3B) seen in 35/63 (55.5%), stapes (Figure 3D) in 30/63 (47.6%), tegmen tympani (Figure 2A) in 24/63 (38%), and facial canal (Figure 3B) in 22/63 (34.9%) out of the diseased temporal bones. The least affected structures were lateral semicircular canal and bony EAC, seen in 3/63 (4.7%) diseased temporal bones each (Table 4).

**Table 3:** HRCT signs of CSOM and cholesteatoma in diseased temporal bones. HRCT: high-resolution computed tomography, CSOM: chronic suppurative otitis media

HRCT signs	Number	Percentage
Loss of aeration and sclerosis of mastoid air cells	61	96.8
Bony erosions	54	85.7
Non-dependent soft tissue density lesions	43	68.2
Bony expansion	35	55.5
Lateral semicircular canal fistula	3	4.7
Petrous Apicitis	1	1.5

**Table 4:** Location of bony erosions in the diseased temporal bones. EAC: external auditory canal

Location of bony erosions	Number	Percentage
Scutum	35	55.5
Malleus	37	58.7
Incus	38	60.3
Stapes	30	47.6
Tegmen tympani thinning/erosion	24	38
Facial canal	22	34.9
Mastoid cortex dehiscence	4	6.3
Lateral semi-circular canal dehiscence	3	4.7
Bony EAC	3	4.7

**Figure 4:**
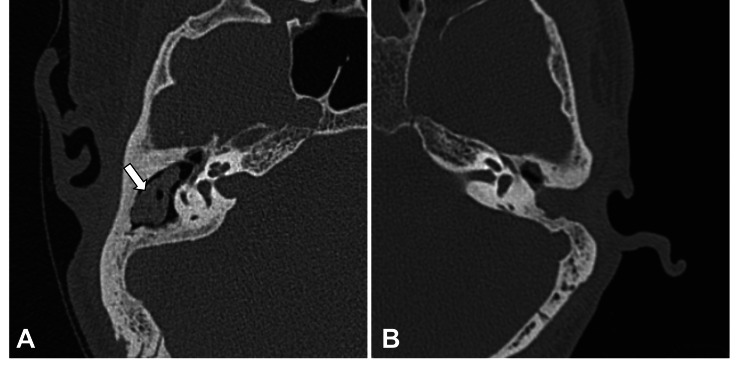
Axial right temporal HRCT image shows soft tissue density in the right post-operative mastoid cavity (A) suspected to be cholesteatoma was proven as wax (white arrow). Axial HRCT image of another patient showing soft tissue density in the left middle ear and post-operative mastoid cavity (B), which was proven to be granulation tissue. HRCT: high-resolution computed tomography

Out of 63 temporal bones with soft tissue density, 58 temporal bones were proven as cholesteatoma, and five were false positive as confirmed on intraoperative/HPE findings. These false-positive cases were granulation tissue (two), cholesterol granuloma (one), non-cholesteatomatous otitis media (one), and wax (one) (Figure 4).

In our study, HRCT temporal bone had a sensitivity of 100% (with CI of 93.8%-100%), specificity of 88.1% (with CI of 74.4%-96.0%), PPV of 92.1% (with CI of 82.4%-97.4%), NPV of 100% (with CI of 90.5%-100%) and accuracy of 95% (with CI of 88.7%-98.4%) for diagnosis cholesteatoma with intra-operative/HPE correlation.

## Discussion

The ability of HRCT to define CSOM and its complication, mainly the cholesteatoma prior to otologic surgery, is well established [7]. HRCT is especially useful in detecting early erosive changes in the ossicles and hidden soft tissue, particularly in the smaller regions like sinus tympani and facial canal recess. HRCT can not characterize and differentiate the cholesteatomatous soft tissue from other pathologies like granulation tissue, scar tissue, wax, granuloma, etc. [6,8]. However, HRCT is superior to magnetic resonance imaging (MRI), positron emission tomography (PET) CT and Technetium-99m studies as it is readily available, economical and gives the detailed bony architecture of the middle ear in the backdrop of CSOM [3]. Though MRI can help characterize the soft tissue, it lacks the spatial resolution and ability to assess bony changes in the temporal bone. 

Out of the 100 temporal bones of the 50 patients studied, 63 temporal bones showed soft tissue density and were labeled as diseased temporal bones on HRCT. Of 63 diseased temporal bones, 54 (85.7%) temporal bones were primarily diseased, and nine (14.3%) temporal bones suspected post-operative recurrence.

In our study, the unilateral disease was seen in 35 (70%) patients, and bilateral disease was seen in 14 (28%) patients, which is in line with the study done by Gomaa et al., which revealed bilateral disease in 16 (28.5%) out of 50 patients [9].

Most of these soft tissue density lesions in our study were non-dependent, i.e., 68.2% of the diseased temporal bones, a vital sign of cholesteatoma. Rai, in their study of 50 patients, reported 45 (90%) patients having non-dependent soft tissue [10]. The different percentage of non-dependent soft tissue in our study was likely due to more extensive soft tissue progression and holotympanic involvement seen in 19 diseased temporal bones.

In the present study, the most common location of soft tissue was the epitympanum/Prussak space seen in 60 (95.2%) of the 63 diseased temporal bones. Other common locations were aditus ad antrum and mesotympanum seen in 51 (80.9%) and mastoid antrum seen in 46 (73%) of the diseased temporal bones. Furthermore, Jacob et al. performed an HRCT study on 30 cases of CSOM with cholesteatoma, which showed soft tissue density in the epitympanum in 22 (73.3%) of patients followed by mesotympanum 17 (56.6%) and aditus ad antrum in 16 (53.3%) of the patients [11].

In the study done by Gomaa et al., according to the typical locations of soft tissue density found in CSOM, the majority of the cases had soft tissue density as extensive holotympanic in 18 (32.1%) patients, followed by attic in 16 (28.5%) patients, attico-antral in 12 (21.4%) and mesotympanum in 10 (17.8%) patients [9]. However, in our study, the most common typical site was attico-antral in 32 (50.7%) diseased temporal bones, followed by extensive holotympanic seen in 19 (30.1%) diseased temporal bones. Attic involvement was seen in 10 (15.8%) diseased temporal bones, and one (1.5%) had mesotympanum and external auditory canal involvement. The EAC cholesteatoma, a rare location for cholesteatoma, was seen in one case and showed surrounding bony erosion, differentiating it from its main differential of keratosis obturans [3].

The loss of aeration and sclerosis of mastoid air cells seen in 61 (96.8%) diseased temporal bones was the most consistent finding of CSOM in our study. In an HRCT evaluation of 64 patients of unsafe type of CSOM by Gaurano et al., 100% of the patients were found to have affected mastoid air cells [12].

Bone erosion was seen in 54 (85.7%) out of 63 diseased temporal bones. Incus was found to be the most common ossicle eroded in 38 (60.3%) diseased temporal bones, followed by malleus in 37 (58.7%) and stapes in 30 (47.6%) diseased temporal bones. A similar trend was reported by Manik et al. in an HRCT study of 50 symptomatic patients, which showed Incus to be the most commonly eroded in 35 (70%) patients, followed by malleus in 21 (42%) and stapes in 17 (34%) patients [13]. Scutum was eroded in 35 (55.5%) diseased temporal bones in the present study. Rai, in their research, reported scutum erosion in 23 (65%) patients [10]. The present research saw tegmen tympani erosion in 24 (38%) diseased temporal bones. In an HRCT study done by Jamal et al. in 50 symptomatic patients, tegmen tympani erosion was seen in 13 (30%) patients [14]. There was no case of dural sinus plate erosion encountered in our study as it is a rare complication of CSOM/cholesteatoma [9-13].

In the present study, bony expansion was seen in 35 (55.5%) diseased temporal bones. However, in the study done by Gaurano et al., bony expansion was seen in 59 (92%) cases [12]. We found the facial canal dehiscence in 22 (34.9%) diseased temporal bones. The study by Jamal et al. reported facial canal dehiscence in 15 (30%) out of 50 patients [14]. In our study, mastoid cortex dehiscence was seen in four (6.3%) diseased temporal bones, similar to Rai, who reported mastoid cortex dehiscence in four (8%) patients [10]. Our study noted lateral semicircular canal fistula in three (4.7%) diseased temporal bones. This observation was seen to concur with an HRCT study done by Dashottar et al. in 50 symptomatic patients, which reported lateral semicircular canal fistula in two (4%) patients [15].

HRCT could detect soft tissue lesions of any size with no false-negative cases in the present study. Hence sensitivity turned out to be 100%. However, five patients were falsely diagnosed with cholesteatoma, as HRCT could not surely differentiate cholesteatoma from granulation tissue (two cases), cholesterol granuloma (one case) and non-cholesteatomatous otitis media (one case). These are some of the common causes for false-positive cases on HRCT as reported in the literature [3,4,6]. One case with soft tissue in the post-operative mastoid cavity suspected as cholesteatoma was found to be impacted wax on surgery. The open mastoid cavity in canal wall down procedures are known to have excessive wax build-up due to altered epithelial migration [16]. We found the specificity of HRCT in diagnosing cholesteatoma to be 88.1% in correlation with the intra-operative and HPE findings. Mitra et al. reported a sensitivity and specificity of 100% for the diagnosis of cholesteatoma in their HRCT study of 100 patients [17]. Payal et al. performed an HRCT temporal bone study on 60 patients and got a sensitivity of 89.6% and specificity of 100% [18]. Reddy et al. found a sensitivity of 92% and a specificity of 66% in their HRCT study of 25 patients of CSOM [19]. 

Limitations

The sample size of our study was only 50 patients. This could be why complications of CSOM like intracerebral complications and dural sinus thrombosis were not seen. Furthermore, the signs of cholesteatoma seen on HRCT, such as mastoid air cells sclerosis, bony erosions, lateral semicircular canal (LSCC) fistula, bony expansion, facial canal, and mastoid cortex dehiscence, were not correlated with intra-operative findings in our study. Thus individual sensitivity and specificity of these findings could not be measured. In addition, 14 patients (28%) had bilateral CSOM disease in our study. Hence, findings in the affected temporal bones were considered instead of affected patients, and due to this, our study could not be classically correlated with other studies which evaluated affected patients. The present study was primarily focussed on the HRCT imaging evaluation of cholesteatoma, and did not assess the underlying pathological mechanism.

## Conclusions

HRCT temporal bone is an invaluable diagnostic tool for detecting early cholesteatoma, tiny and cholesteatoma in hidden locations. It helps assess the bony integrity, detects various associated complications with high accuracy, and provides a roadmap for the surgeon during cholesteatoma surgery. In addition, HRCT also helps assess the recurrent disease in the post-operative temporal bones. Thus, HRCT provides a diagnostic capability with exceptional sensitivity and considerably high specificity in detecting cholesteatoma and the state of the bone structures in the temporal bone.
